# *Announcement: *National Kidney Month — March 2017

**DOI:** 10.15585/mmwr.mm6608a7

**Published:** 2017-03-03

Each year, March is designated National Kidney Month to raise awareness about the prevention and early detection of kidney disease. In the United States, kidney disease is the ninth leading cause of death (*1*). Approximately one in seven (15%) U.S. adults aged ≥20 years are estimated to have chronic kidney disease, most of whom are unaware of their condition (*2*). If left untreated, chronic kidney disease can lead to kidney failure, requiring dialysis or transplantation for survival (*3*).

Risk factors for chronic kidney disease include diabetes, high blood pressure, cardiovascular disease, and obesity (*3*), and controlling diabetes and high blood pressure can delay or prevent chronic kidney disease and improve health outcomes (*3*). Lifestyle changes to increase physical activity, improve nutrition, and lose weight have been shown to prevent or delay type 2 diabetes among persons at risk (*4*), and might offer the greatest benefit in preventing chronic kidney disease.

In collaboration with partners, CDC supports and maintains the Chronic Kidney Disease Surveillance Project website (https://www.cdc.gov/ckd/surveillance) to document and monitor the burden of chronic kidney disease and its risk factors in the U.S. population and to track progress in chronic kidney disease prevention, detection, and management (*2*). Information is available about kidney disease prevention and control at https://www.nkdep.nih.gov and about diabetes prevention and control at https://www.cdc.gov/diabetes.
